# Duplication and Diversification of the Hypoxia-Inducible IGFBP-1 Gene in Zebrafish

**DOI:** 10.1371/journal.pone.0003091

**Published:** 2008-08-28

**Authors:** Hiroyasu Kamei, Ling Lu, Shuang Jiao, Yun Li, Claus Gyrup, Lisbeth S. Laursen, Claus Oxvig, Jianfeng Zhou, Cunming Duan

**Affiliations:** 1 Department of Molecular, Cellular, and Developmental Biology, University of Michigan, Ann Arbor, Michigan, United States of America; 2 Laboratory of Molecular Medicine, School of Medicine and Pharmacy, Ocean University of China, Qingdao, China; 3 Department of Molecular Biology, University of Aarhus, Aarhus C, Denmark; Katholieke Universiteit Leuven, Belgium

## Abstract

**Background:**

Gene duplication is the primary force of new gene evolution. Deciphering whether a pair of duplicated genes has evolved divergent functions is often challenging. The zebrafish is uniquely positioned to provide insight into the process of functional gene evolution due to its amenability to genetic and experimental manipulation and because it possess a large number of duplicated genes.

**Methodology/Principal Findings:**

We report the identification and characterization of two hypoxia-inducible genes in zebrafish that are co-ortholgs of human IGF binding protein-1 (IGFBP-1). IGFBP-1 is a secreted protein that binds to IGF and modulates IGF actions in somatic growth, development, and aging. Like their human and mouse counterparts, in adult zebrafish *igfbp-1a* and *igfbp-1b* are exclusively expressed in the liver. During embryogenesis, the two genes are expressed in overlapping spatial domains but with distinct temporal patterns. While zebrafish IGFBP-1a mRNA was easily detected throughout embryogenesis, IGFBP-1b mRNA was detectable only in advanced stages. Hypoxia induces *igfbp-1a* expression in early embryogenesis, but induces the *igfbp-1b* expression later in embryogenesis. Both IGFBP-1a and -b are capable of IGF binding, but IGFBP-1b has much lower affinities for IGF-I and -II because of greater dissociation rates. Overexpression of IGFBP-1a and -1b in zebrafish embryos caused significant decreases in growth and developmental rates. When tested in cultured zebrafish embryonic cells, IGFBP-1a and -1b both inhibited IGF-1-induced cell proliferation but the activity of IGFBP-1b was significantly weaker.

**Conclusions/Significance:**

These results indicate subfunction partitioning of the duplicated IGFBP-1 genes at the levels of gene expression, physiological regulation, protein structure, and biological actions. The duplicated IGFBP-1 may provide additional flexibility in fine-tuning IGF signaling activities under hypoxia and other catabolic conditions.

## Introduction

The evolution of gene function is a fundamental issue in modern biology. Gene duplication is considered to be the primary force of new gene evolution, and is evident in a number of sequenced genomes ranging from Bacteria to human [1]. Divergent functions in duplicates might help permanent preservation of the genes. Two major hypotheses have been advanced to explain the functional retention of the duplicated genes. The neofunctionalization hypothesis argues that after duplication one daughter gene retains the ancestral function while the other acquires new functions [2]. The duplication-degeneration-complementation (DDC) hypothesis asserts that the functions of the ancestral gene are partitioned between the duplicated genes, such that the duplicate genes complement each other by jointly performing the necessary subfunctions of the ancestral gene [3]. These two hypotheses may not necessarily be mutually exclusive. It has been proposed recently that a large portion of duplicated genes undergo rapid subfunctionalization followed by prolonged and substantial neofunctionalization [4].

Despite the advance in our understanding of the role of gene duplication in the evolution of new genes and gene families, deciphering whether a pair of duplicated genes has evolved divergent functions is often challenging. The zebrafish is uniquely positioned to provide insight into the process of functional gene evolution due to its versatility, amenability to genetic and experimental manipulation, and because it possess a large number of duplicated genes. By one estimation, zebrafish has two co-orthologs for at least 20% of human genes [5]. Recent studies have suggested that a genome duplication event occurred in the teleost lineage ∼350 million years ago, prior to the beginning of the teleost radiation [5], [6].

We have previously reported the isolation of *igfbp-1* (insulin-like growth factor binding protein-1) that is expressed in the liver in zebrafish [7] and plays an important role in mediating hypoxia-induced embryonic growth and developmental delay [8]. Here, we describe a new and related zebrafish gene. Phylogenetic, gene structure, and chromosomal location analysis indicate this gene is an *igfbp-1* paralog and therefore we termed it as *igfbp-1b* and the previously reported gene as *igfbp-1a*. Both genes encode proteins that are related to IGFBP-1 reported in humans and other mammals. IGFBP-1 is a secreted protein belonging to the IGFBP gene family [9], [10], [11]. The IGF signaling pathway is an evolutionarily conserved pathway that plays important roles in regulating growth, development, reproduction, and aging [12], [13]. IGFBPs selectively bind IGF with high affinities and modulate IGF interaction with the cell surface IGF-1 receptor (IGF-1R) [9], [10], [14]. Like other IGFBPs, IGFBP-1 has conserved N-terminal and C-terminal domains and a variable linker (L)-domain. Its expression is highly inducible under a variety of catabolic conditions such as food deprivation, malnutrition, stress, injury, and hypoxia [11].

To understand whether the duplicated IGFBP-1 genes have evolved distinct structural and functional properties, we have investigated their spatial and temporal expression, physiological regulation, IGF binding kinetics, and biological activities. These results indicate that the two IGFBP-1 genes have gone through subfunction partitioning at the temporal expression, physiological regulation, protein structure and functional levels and provide novel insights into the functional conservation and divergence of the duplicated *igfbp-1* genes.

## Results

### Identification of a new zebrafish IGFBP gene

**Figure 1:**
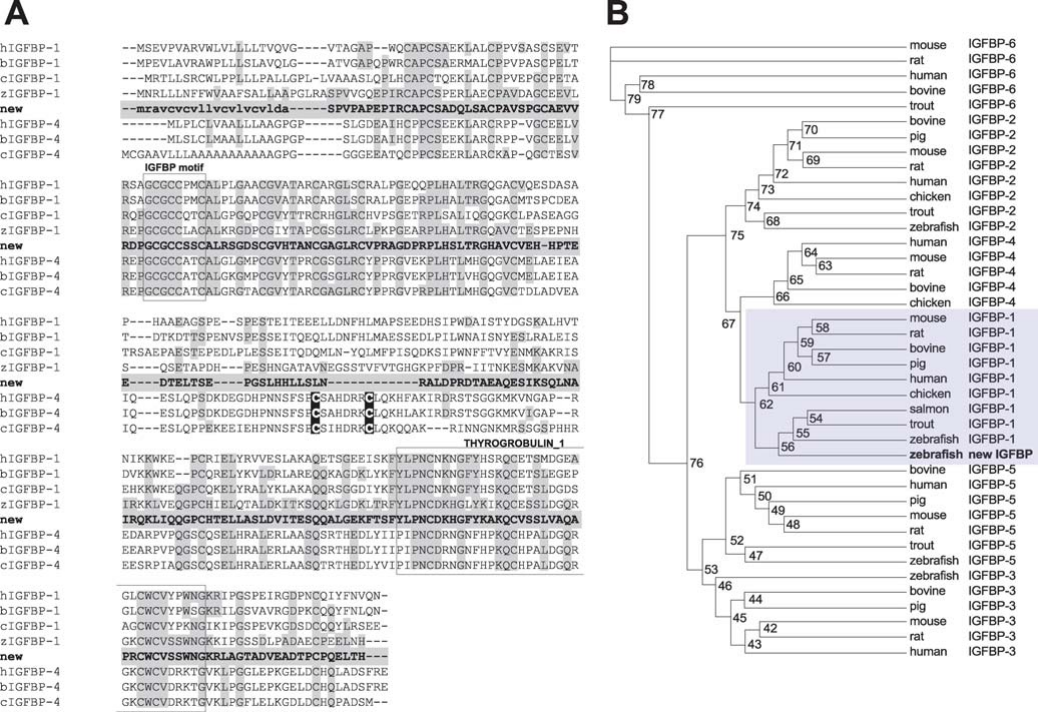
Structural and phyologenetic analysis. A) Comparison of the new zebrafish IGFBP primary sequence with that of human (h), bovine (b), chicken (c), and zebrafish (z) IGFBP-1 and -4. Amino acids identical with the new IGFBP are light-shaded. The putative signal sequence of the new IGFBP is shown in small letters. The IGF-binding motif and thyroglobulin-1 motif conserved among all known IGFBPs are shown in open boxes. The two cystein residues characteristically located in the L-domain of IGFBP-4 are dark-shaded. B) Phylogenetic tree of the IGFBP gene family. Amino acid sequences of full-length IGFBPs were analyzed using the Maximum Likelihood method. Values on branches are percentages of times that the two clades branched as sisters (1000 run). The results indicate that the newly identified IGFBP is most closely related to those in the IGFBP-1 subgroup.

In searching IGFBP genes in zebrafish, we have identified a new IGFBP like gene (ENSDARG00000038666, DQ465370) in the zebrafish genome database. Using 5′- and 3′-RACE, its full-length cDNA was determined (DQ465369). The encoded protein (ABE98257) shares higher identities with IGFBP-1 and -4 than with other known IGFBPs. Structural analysis suggests that the mature peptide is composed of 224 aa with a predicted molecular mass of 23.8 kDa. Like other known IGFBPs, there are 12 cysteine residues in its N-terminal domain (residues 1–82) and 6 cysteine residues in its C-terminal domain (residues 156–244; Fig. 1A). A typical IGFBP motif is present in the N-domain and a thyroglobulin-1 motif in the C-domain. As shown in Table 1, the overall sequence identities of this protein with six human IGFBPs are 35% (IGFBP-1), 28% (IGFBP-2), 28% (IGFBP-3), 33% (IGFBP-4), and 30% (IGFBP-5 and IGFBP-6). Its identities with the known zebrafish IGFBPs are 50% (IGFBP-1), 28% (IGFBP-2), 26% (IGFBP-3), and 30% (IGFBP-5), respectively. If only the N- and C-domains were used for comparison, the sequence identities were much higher. Among various members of the IGFBP family, the new IGFBP has higher sequence identities with IGFBP-1s and IGFBP-4s. IGFBP-4s are unique among the IGFBP family in that they have two extra cysteine residues in the L-domain. No cysteine is found in the L-domain of this newly identified protein (Fig. 1A). In the L-domain of mammalian IGFBP-1, there is a proline, glutamine, serine, and threonine (PEST) motif, known to contribute to its susceptibility to proteolysis [15]. The PEST sequence is present in this protein. Mammalian IGFBP-1 has an Arg-Gly-Asp (RGD) motif in its C-domain, but it is not found in the previously reported zebrafish IGFBP-1 [7]. Likewise, this new IGFBP does not contain any RGD motif (Fig. 1A).

**Table 1:** Sequence identities between the novel zebrafish IGFBP and other IGFBPs.

Species†/Protein	IGFBP-1	IGFBP-2	IGFBP-3	IGFBP-4	IGFBP-5	IGFBP-6
human	35	28	28	33	30	30
mouse	34	27	30	33	32	27
rat	33	27	28	33	31	27
pig	36	27	30	-	31	-
bovine	37	29	28	33	25	26
chicken	35	27	-	35	-	-
frog	33	-	-	32*	30	-
salmon	55	-	-	-	-	-
trout	55	27	25	24*	31	25
fugu	56*/55*/50*	28*/28*	30	33	30	-
tetraodon	32	30*/29	31/28*	-	36*	27
zebrafish	50	28	26	-	30	-

† human (*Homo sapiens*), mouse (*Mus musculus*), rat (*Rattus norvegicus*), pig (*Sus scrofa*), bovine (*Bos taurus*), chicken (*Gallus gallus*), frog (*Xenopus laevis*), salmon (*Oncorhynchus tshawytscha*), trout (*Oncorhynchus mykiss*), fugu (*Takifugu rubripes*), tetraodon (*Tetraodon nigroviridis*), zebrafish (*Danio rerio*).

* Compared with corresponding partial sequences.

### The newly identified gene is a co-ortholog of IGFBP-1 gene

We next performed phylogenetic analysis using full-length amino acid sequences of all known IGFBPs. In the PlotTest program, the best model (JTT+G+I) was selected and the tree was generated using PHYML program. Although the new IGFBP shares similarly high sequence identities with human IGFBP-1 and IGFBP-4, phylogenetic analyses grouped it into the IGFBP-1 clade (Fig. 1B), indicating that it is a paralog or co-ortholog of human IGFBP-1. We therefore named the previously reported zebrafish IGFBP-1 gene [7] as *igfbp-1a*, and this new gene as *igfbp-1b*.

**Figure 2:**
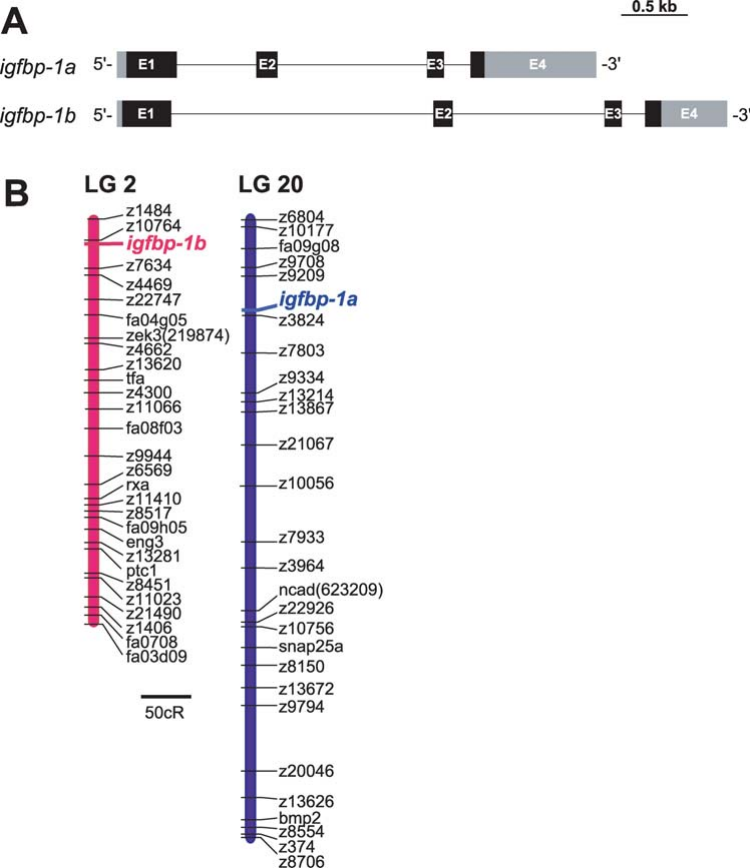
Genomic structure and chromosomal location of zebrafish *igfbp-1a* and *igfbp-1b*. A) A schematic diagram comparing the genomic structures of *igfbp-1a* and *-1b*. Exons are shown as boxes and introns shown as lines. Lightly and darkly shaded areas represent untranslated region (UTR) and open reading frame (ORF), respectively. The zebrafish *igfbp-1a* structure is taken from Kajimura et al. (2005). Scale bar is 0.5 kb. B) Zebrafish *igfbp-1a* is located on linkage group (LG) 20, 32.16 centiRay (cR) from marker z9209; *igfbp-1b* is located on LG2, 10.65cR from marker z10764, respectively. 1cR = 148 kb. Scale is 50 cR.

The genomic structure of *igfbp-1b* was determined by searching the zebrafish genome database and genomic PCR analysis. *igfbp-1b* is 4,198 bps long and contains 4 exons and 3 introns (Fig. 2A). The size of *igfbp-1b* and its overall structure are similar to those of *igfbp-1a* [8] (Fig. 2A). We mapped the chromosomal loci of *igfbp-1a* and *igfbp-1b* using the LN54 radiation hybrid panel. While *igfbp-1a* is located to linkage group LG 20, *igfbp-1b* is mapped to LG 2 (Fig. 2B). Further analysis suggested that zebrafish *igfbp-1a* and five neighboring genes (*LOC563662*, *ccm2*, *igfbp-3*, *LOC562593* and *zp3a*) have orthologs locating on human chromosome 7 (Table 2). The human IGFBP-1 gene resides on chromosome 7 next to the IGFBP-3 gene in a tail-to-tail fashion [16], [17]. Likewise, zebrafish *igfbp-1a* and *igfbp-3* were found to be next to each other on LG 2, in a tail-to-tail fashion. In the proximate region, there are five other zebrafish genes (*eng3a*, *fa04b06d*, *grb10*, *inhibal* and *twhhb*), whose orthologs are located on human chromosome 7. These results suggest that *igfbp-1a* and *igfbp-1b* have syntenic correspondence with the human IGFBP-1 gene.

**Table 2:** Genes located on zebrafish LGs 2 and 20 and those located on human chromosome 17 and 7.

*zebrafish LG.2*	*human chr. 17*	*zebrafish LG.20*	*human chr. 7*
*AI477439.1*	*QRICH2*		
*zgc:77429*	*EIF5A*		
*Dr.104464*	*LOC342541*		
*igfbp1b*	*IGFBP4*		
*BE201786.1*	*GFAP*		
		*LOC563662*	*CAMK2B*
		*ccm2*	*CCM2*
		*igfbp1a*	*IGFBP1*
		*igfbp3*	*IGFBP3*
		*LOC562593*	*KBTBD2*
		*zp3a*	*ZP3*
*eng3a*			*EN2*
*fa04b06d*			*CAGF28*
*grb10*			*GRB10*
*inhbal*			*INHBA*
*twhhb*			*SHH*

### The duplicated igfbp-1 genes exhibit overlapping and distinct expression patterns and are regulated by nutritional state

**Figure 3:**
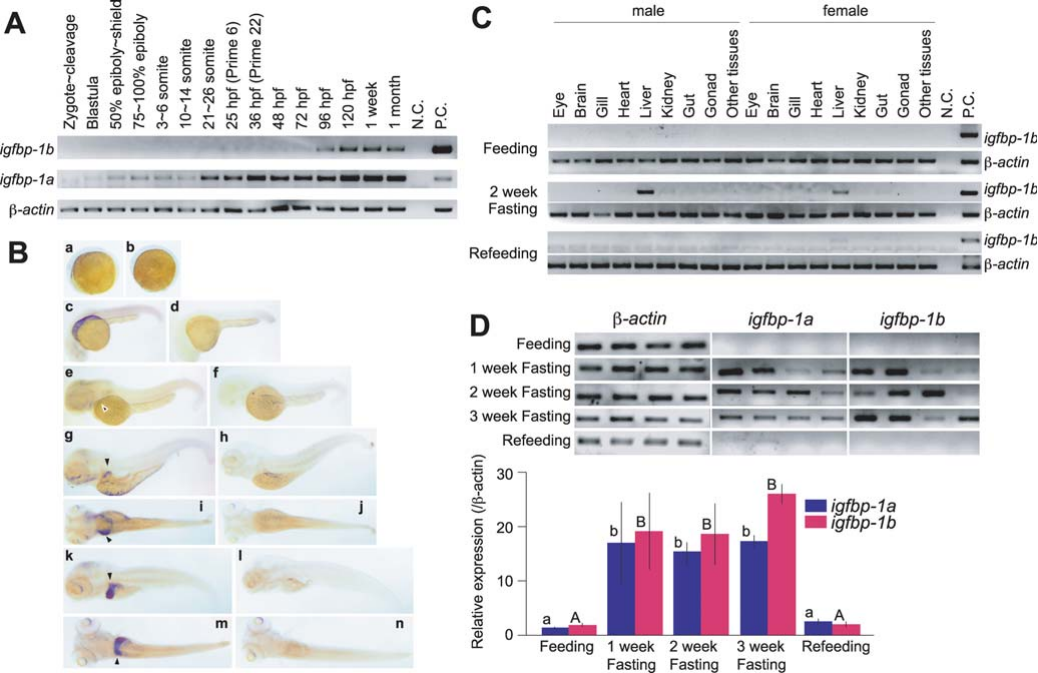
Temporal and spatial expression patterns of *igfbp-1a* and *igfbp-1b* during zebrafish embryogenesis. A) RT-PCR analysis result. The developmental stages are shown at the top, hpf, hour post fertilization. B) *In situ* hybridization analysis of IGFBP-1b mRNA. Embryos of 5-somite (a, b), prim.5 straightening period (c, d), the long bud stage (e, f), 120 hpf hatched embryos (g, h, I, j) and 196 hpf hatched embryos (k, l, m, n) were analyzed. Shown in panels a, c, e, g, i, k and m are embryos probed with the antisense riboprobe. In panels b, d, f, h, j, l and n, embryos probed with sense riboprobe are shown. Panels a–f, k and l are lateral views with the head to the left, and panels i, j, m and n are corresponding dorsal views of panels g, h, k and l, respectively. IGFBP-1b mRNA signals were weakly detectable in the liver at 48 hpf, and highly expressed in the liver of 120 and 196 hpf embryos (indicated by arrowheads). C) Tissue distribution of *igfbp-1b* mRNA in male and female adult fish. Matured adult male and female fish were sampled at the beginning of the experiment (Feeding), after fasting for 2 weeks (2 week Fasting) or 2 week fasting followed by 1 week re-feeding (Refeeding). RNA was extracted from various tissues and subjected to RT-PCR analysis. D) Nutritional regulation of IGFBP-1a and -1b mRNA levels in adult zebrafish.Total RNA was isolated from adult fish at the beginning of the experiment (Feeding), after fasting for 1 week (1 week Fasting), 2 weeks (2 week Fasting), 3 weeks (3 week Fasting), or refed for a week after a 2 week fasting (Refeeding). RNA was subjected to RT-PCR analysis and a representative RT-PCR result is shown in the upper panel. The relative levels of IGFBP-1a and -1b mRNA were measured and normalized by corresponding b-actin signals and shown in the bottom panel. Values are mean±S.E. (n = 4). Values marked with different letters (a and b, or A and B) are significantly different from each other (p<0.05).

The spatial and temporal expression patterns of *igfbp-1a* were determined and compared to those of *igfbp-1b*. As shown in Fig. 3A, while zebrafish IGFBP-1a mRNA was easily detected throughout embryogenesis, IGFBP-1b mRNA was not detectable until the prime stage (25 hpf) at low levels. This lasted until 72 hpf. IGFBP-1b mRNA became easily detectable at 96 hpf larval stage and thereafter. Whole mounted *in situ* hybridization analysis results of IGFBP-1a mRNA expression has been published [7]. In agreement with the RT-PCR data, whole mounted *in situ* hybridization analysis showed that IGFBP-1b mRNA became detectable at 48 hpf in primordial hepatic tissues (Fig. 3B, panel e) and the hepatic expression was clearly detected at 72, 96, and 120 hpf (Fig. 3B, panels g, i, k and m). The hybridization signal was specific because the corresponding sense RNA probe did not give any positive signals in the same batch of embryos (Fig. 3B, panels b, d, f, h, j, l, and n).

It has been previously shown that the zebrafish *igfbp-1a* is expressed in adult liver in fasted but not well fed animals [7]. We therefore analyzed the expression of zebrafish IGFBP-1b mRNA in various adult tissues of normally fed as well as 2-week fasted animals. As shown in Fig. 3C, IGFBP-1b mRNA was not detected in fed animals. Two week food deprivation strongly induced the hepatic IGFBP-1b mRNA expression in both male and female adult fish. No IGFBP-1b mRNA was detectable in eye, brain, gill, heart, kidney, gut, ovary, testes and other remaining tissues. Re-feeding for 1 week reduced IGFBP-1b mRNA to undetectable levels (Fig. 3C), indicating *igfbp-1b* is also expressed in adult liver and its expression is induced by starvation.

To compare the effect of starvation on IGFBP-1a and -1b mRNA expression, adult zebrafish were deprived of food for up to 3 weeks. As shown in Fig. 3D, the levels of IGFBP-1a and -1b mRNA in normal, well-fed adult animals were very low. One week fasting increased the IGFBP-1a and -1b mRNA level by 17∼20-fold (p<0.05). This induction maintained throughout the 3-week period. Compared to the levels of IGFBP-1a, the IGFBP-1b levels appeared higher at the end of the third week. Re-feeding of the fasted fish for 1 week reduced the IGFBP-1a and -1b mRNA levels to the basal level. No significant change was seen in the levels of β-actin mRNA (Fig. 3D). These data suggest that *igfbp-1a* and *igfbp-1b* are expressed in overlapping spatial domain(s) during embryogenesis but they exhibit distinct temporal expression pattern. In adult stages, both *igfbp-1* genes are only expressed in the liver and their expression levels are regulated by nutritional status.

### The two igfbp-1 genes are differentially regulated by hypoxia

**Figure 4:**
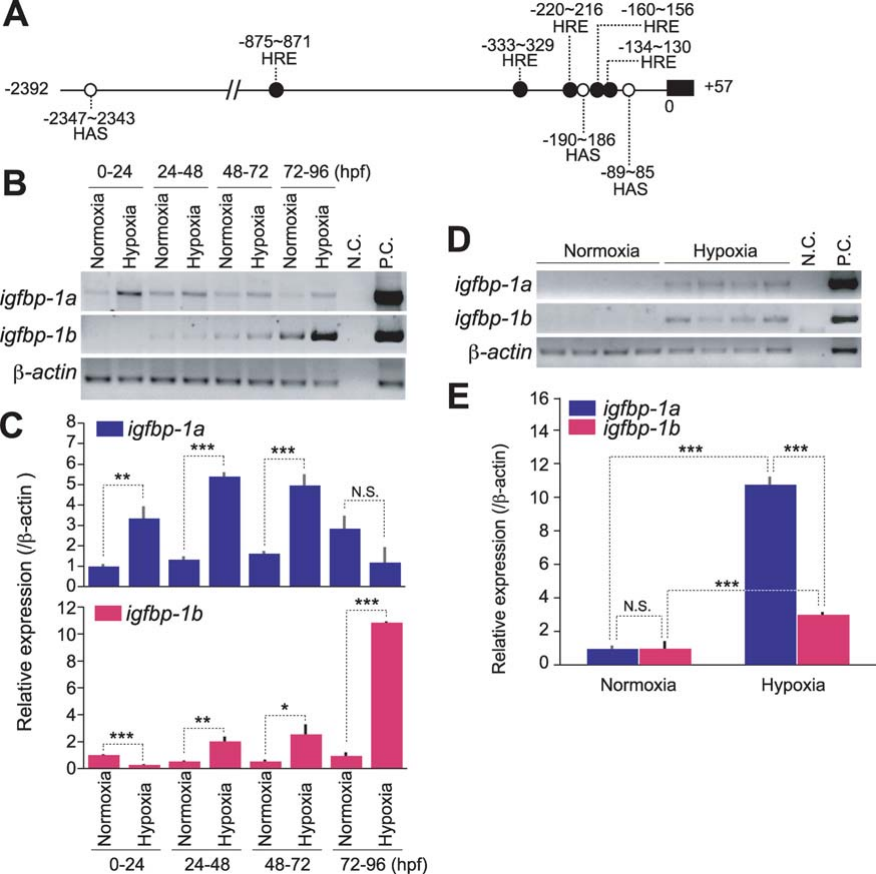
The expression levels of *igfbp-1a* and *igfbp-1b* are differentially regulated by hypoxia. A) Schematic diagram of the 5′-flanking region of *igfbp-1b*. 2,347 bp before the start codon are shown. 5 hypoxia response elements (HRE: •) and 3 hypoxia inducible factor-1 (HIF-1) ancillary sequences (HAS: ○) are found in this region. B,C) Effects of hypoxia in developing zebrafish embryos. 0, 24, 48, and 72 hpf embryos were subjected to 24 h hypoxia as described in *Materials and Methods*. Total RNA was isolated and subjected to RT-PCR and qRT-PCR analysis. Representative RT-PCR results are shown in the upper panel B). IGFBP-1a and -1b mRNA levels were further analyzed by qRT-PCR and are expressed as relative value to those of the normoxia groups C). Data shown are means±S.E. of three independent experiments. *p<0.05, **p<0.01 compared to the normoxia group at the same time point. D, E) Hypoxia increases IGFBP-1a and -1b mRNA levels in adult fish. Total RNA was isolated from individual zebrafish that were kept in normaxia or intermediate hypoxia for 6 h. Representative RT-PCR results are shown in the upper panel D). qRT-PCR data are expressed as relative values to those of the normoxia group and shown in the lower panel E). Data shown are means±S.E. of three independent experiments. *p<0.05, **p<0.01 compared to each group.

Hypoxia has been shown to strongly induce zebrafish *igfbp-1a* gene expression via activating the HIF-1 pathway during embryogenesis and in adult stage [7], [8]. Sequence analysis of the 2.3 kb region upstream of the translational start site in *igfbp-1b* revealed the presence of 5 putative hypoxia response elements (HREs) and 3 possible HIF-1 ancillary sequences (HAS) (Fig. 4A), indicating possible involvement of hypoxia/HIF-1 in regulating *igfbp-1b* expression. To examine whether and when hypoxia induces *igfbp-1b* expression, embryos of various stages (0, 24, 48 and 72 hpf) were subjected to hypoxia treatment for 24 h. As shown in Fig. 4B, 24 h hypoxia treatment of 0, 24, and 48 hpf embryos resulted in a marked increase in IGFBP-1a mRNA levels. The effect was not seen in embryos of advanced stage (72–96 hpf). In contrast, hypoxia had little or only modest effect on the IGFBP-1b mRNA expression in early embryos (0 and 24 hpf), but it had a strong effect in later stage (72 and 96 hpf). Further qRT-PCR analysis indicated that 24 h hypoxia treatment of 0∼4 hpf embryos resulted in a 3.4 fold increase (p<0.01) in IGFBP-1a mRNA levels, while it caused a 3.7 fold decrease (p<0.001) in IGFBP-1b mRNA levels (Fig. 4C). Twenty four hour hypoxia treatment of 24 and 48 hpf embryos resulted in 3.4∼5.5 fold, highly significant increases (p<0.001) in IGFBP-1a mRNA levels and modest but statistically significant increases in IGFBP-1b mRNA levels (2.1 and 2.6 fold increases, p<0.01∼0.05). A 10.9 fold highly significant (p<0.001) increase was detected in the IGFBP-1b mRNA levels in 72 hpf, while there was no increase in the IGFBP-1a mRNA levels at this stage.

Under normoxia and well-fed condition, both IGFBP-1a and -1b mRNA levels were very low in adult fish, undetectable by RT-PCR (Fig. 4D). Hypoxia strongly induced the expression of both genes (Fig. 4D). qRT-PCR analysis results indicated that 24 h hypoxia treatment resulted in a 10.7 fold increase (p<0.01) in IGFBP-1a mRNA levels, and a 3.0 fold increase (p<0.01) in IGFBP-1b mRNA levels (Fig. 4E). Together, these data suggest that while the expression of both *igfbp-1a* and *igfbp-1b* is induced by hypoxia during embryogenesis and in adult stages, there are clear differences in the timing and responsiveness of these two genes to hypoxia. Hypoxia strongly induces the *igfbp-1a* expression in early embryogenesis, but it preferentially induces the *igfbp-1b* expression later in embryogenesis. In adult stages, hypoxia has a stronger effect on *igfbp-1a* than on *igfbp-1b*.

### IGFBP-1a and -1b proteins have distinct IGF binding kinetics

**Figure 5:**
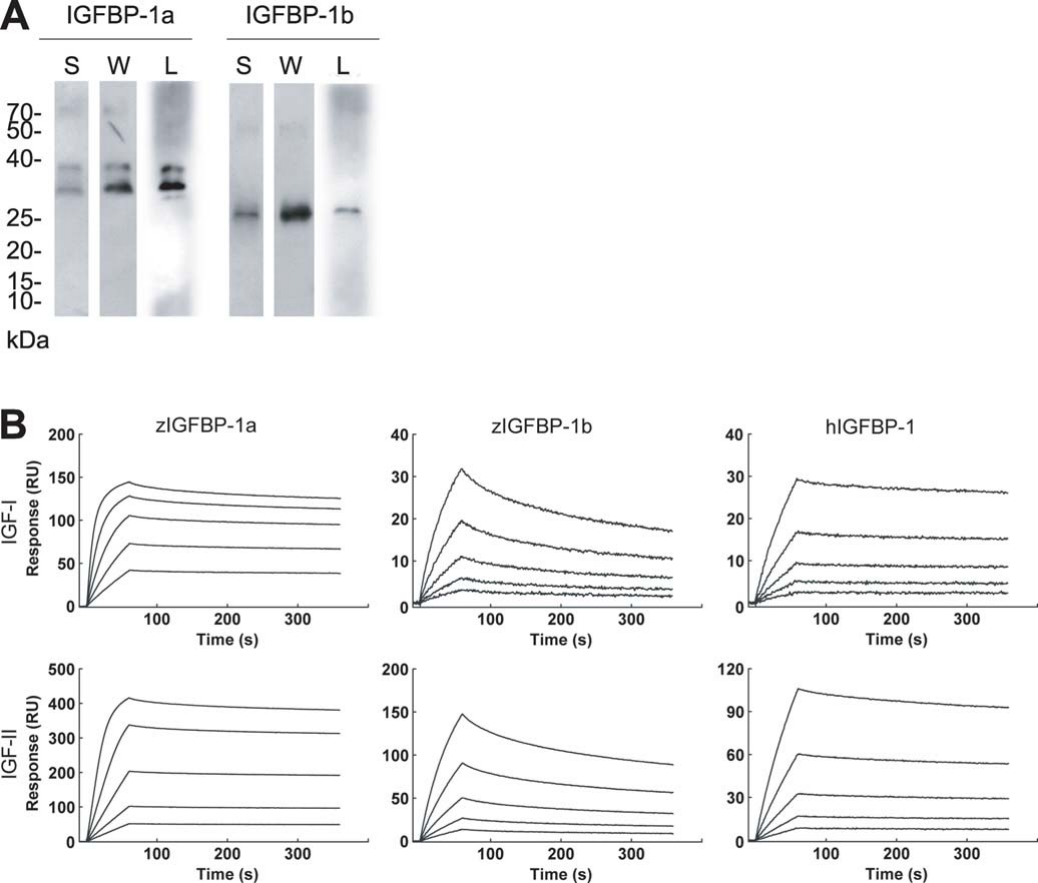
Zebrafish IGFBP-1a and -1b proteins have different IGF binding affinities and kinetics. A) Purification and characterization of zebrafish IGFBP-1a and -1b proteins. Isolated IGFBP-1a and -1b proteins were analyzed by SDS-PAGE under non-reducing condition, followed with Silver staining (S), western blotting (I) and western ligand-blotting (L). 1.0 (S) or 0.3 µg (W and L) purified protein was loaded to each lane. (B) BIAcore analysis of IGFBP1a and -1b. All binding experiments were carried out at 25°C in HBS buffer with a constant flow rate of 30 µL/min. Different concentrations of zebrafish IGFBP-1a and -1b or human IGFBP-1 were injected over the surface for 60 seconds followed by a five minute dissociation period. The sensor surface was regenerated by two 30 second injections of HCl (100 mM). Sensorgram data were analyzed using the BIAcore T100 evaluation Software version 1.1. Concentrations of IGFBP-1a applied were 144 nM, 72 nM, 36 nM, 18 nM and 9 nM, IGFBP-1b were 173 nM, 86.4 nM, 43.2 nM, 21.6 nM and 10.8 nM and human IGFBP-1 were 368 nM, 184 nM, 92 nM, 46 nM and 23 nM.

To study the biological actions of the two zebrafish IGFBP-1s, recombinant IGFBP-1a, -1b, and human IGFBP-1 were produced in HEK293t cell and purified. The two zebrafish proteins had different apparent sizes on SDS-PAGE. IGFBP-1a showed as a doublet and had a greater apparent size (31.7 and 28.7 kDa), whereas IGFBP-1b was smaller (26.8 kDa; Fig. 5A). Ligand blotting showed that both IGFBP-1a and -1b proteins are indeed functional IGF binders (Fig. 5A). The binding kinetics for their interactions with IGF-I and -II were further analyzed by surface plasmon resonance (Fig. 5B and Table 3). Human IGFBP-1 showed similar high-affinity binding to IGF-I and -II (*K_D_* = 5.96 and 8.63 nM). Zebrafish IGFBP-1a binds with very high-affinity to IGF-I (*K_D_* = 0.28 nM) and IGF-II (*K_D_* = 0.34 nM). In both cases, a very fast association rate (IGF-I, *k_a_* = 11.80±0.04×10^5^ M^−1^ s^−1^; IGF-II, *k_a_* = 8.67±0.08×10^5^ M^−1^ s^−1^) and a slow dissociation rate (*k_d_* IGF-I, 3.32±0.007×10^−4^ s^−1^; *k_d_* IGF-II, 2.90±0.007×10^−4^ s^−1^) were found, suggesting the existence of highly stable IGFBP-1a/IGF complexes. The dissociation constants for the interactions between zebrafish IGFBP-1b and IGF ligands were *K_D_* = 12.0 nM (IGF-I) and *K_D_* = 9.33 nM (IGF-II), which are respectively 43 and 27 fold lower compared with those of zebrafish IGFBP-1a. Interestingly, IGFBP-1b retains a relatively higher association rate with IGF-I (*k_a_* = 1.71±0.004×10^5^ M^−1^ s^−1^) and a remarkably higher association rate with IGF-II (*k_a_* = 9.93±0.002×10^5^ M^−1^ s^−1^). However, these complexes were much less stable with very high dissociation rates (*k_d_* IGF-I, 20.60±0.0053×10^−4^ s^−1^; *k_d_* IGF-II, 92.60±0.32×10^−4^ s^−1^). These data suggest that the two zebrafish proteins have very different IGF binding properties: IGFBP-1b has much lower affinities to IGFs compared with IGFBP-1a.

**Table 3:** Kinetic analysis of zebrafish IGFBP-1a, -1b and human IGFBP-1 interactions with IGF biosensor surfaces.

IGF-1					
	*k_a_*(×10^5^ M^−1^×s^−1^)	*k_d_*(×10^−4^ s^−1^)	*K_D_*(nM)	χ^2^	Relative *K_D_*
zebrafish IGFBP-1a	11.80±0.040	3.32±0.007	0.28	0.2	1.0
zebrafish IGFBP-1b	1.71±0.004	20.60±0.053	12.0	0.202	42.8
human IGFBP-1	0.53±0.001	3.14±0.008	5.96	0.032	21.2

IGF-2					
	*k_a_*(×10^5^ M^−1^×s^−1^)	*k_d_*(×10^−4^ s^−1^)	*K_D_*(nM)	χ^2^	rekative *K_D_*
zebrafish IGFBP-1a	8.67±0.08	2.9±0.007	0.34	3.28	1.0
zebrafish IGFBP-1b	9.93±0.002	92.60±0.320	9.33	1.26	27.4
human IGFBP-1	0.53±0.002	4.57±0.009	8.63	0.082	25.5

Association rate (*k_a_*), dissociation rate (*k_d_*), and dissociation constants (*K_D_*: *k_d_/k_a_*) are given.

### IGFBP-1a and -1b have different activity in inhibiting IGF actions

**Figure 6:**
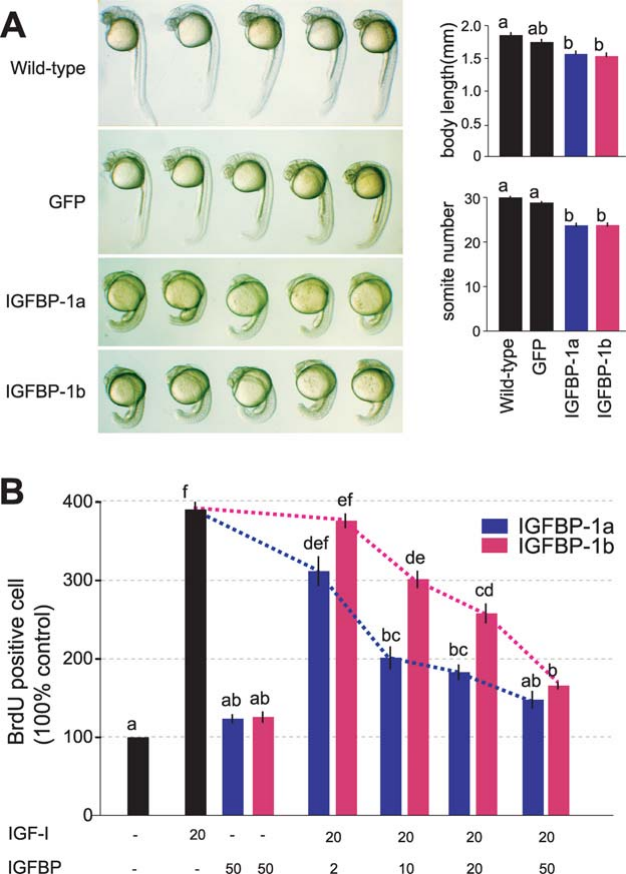
Zebrafish IGFBP-1a and IGFBP-1b are both functional but have different activities. A) Expression of IGFBP-1a and -1b reduces embryo growth and developmental rate in vivo. GFP mRNA (600 pg/embryo), a mixture of IGFBP-1a mRNA (400 pg/embryo) and GFPmRNA (200 pg/embryo), or IGFBP-1a mRNA (400 pg/embryo) and GFP mRNA (200 pg/embryo) was injected into 1–2 cell stage embryos. The embryos were raised to 24 hpf and body length and somite number were determined. Representative embryos are shown in the left column. Body length and somite number of each experimental group are shown in the left column. Values are represented as means±S.E. (n = 21–29). Groups with common letters are not significantly different from each other (p<0.05). B) Effects of IGFBP-1a and -1b on IGF-I-stimulated cell proliferation in cultured zebrafish cells. Serum starved confluent cells were exposed to the media with or without 20 nM IGF-I in the absence or presence of various concentrations of IGFBP-1s (2, 10, 20 or 50 nM). To evaluate IGF-independent effect, cells are exposed with 50 nM of IGFBP-1s without IGF. The percentage of BrdU-positive cells in each group was calculated and is shown. Values are means±S.E. (n = 3∼6). Groups with common letters are not significantly different from each other (p<0.05).

It has been shown that IGFBP-1a inhibits zebrafish embryo development by binding to IGFs and sequestering their binding to the IGF-I receptor [8]. To determine the biological actions of IGFBP-1b *in vivo*, capped IGFBP-1b mRNA was co-injected with GFP mRNA to zebrafish embryos at 1–2 cell stage. Capped IGFBP-1a mRNA was used as a positive control and GFP mRNA alone as a negative control. As shown in Fig. 6A, embryos injected with GFP mRNA alone were indistinguishable in morphology to those of wild-type embryos. There was no notable difference in either body length or somite number (Fig. 6A, left panel). Compared to wild-type or the GFP mRNA group, injection of IGFBP-1a or -1b mRNA resulted in a significant reduction (p<0.05) in somite number, suggesting a delay in the developmental rate. The mean body size of IGFBP-1a and -1b mRNA injected embryos was also significantly smaller than those of wild-type embryos (*p*<0.05, Fig. 6A).

To determine and compare the biological activities of zebrafish IGFBP-1a and -1b in modulating IGF actions, purified IGFBP-1a or -1b was added to cultured zebrafish embryonic cells (ZF4) in the presence of absence of IGF-I and BrdU incorporation rates were measured. Exposure of ZF-4 cells to IGF-I at 20 nM resulted in a 372.8±10.2% increase in BrdU labeling index (p<0.05, *n* = 6). IGFBP-1a, when added together with IGF-1, inhibited IGF-I-stimulated DNA synthesis in a concentration-dependent manner. At 10 nM (1∶2 molar ration to IGF-I), IGFBP-1a caused a significant decease in IGF-I action (Fig. 6B). At the highest concentration (50 nM), IGFBP-1a reduced the DNA synthesis close to the basal level (148.8±11.5% of control), suggesting a near complete inhibition of IGF-I actions. Consistent with the IGF binding affinity results, IGFBP-1b had a weaker effect. At 10 nM, IGFBP-1b caused a 22.7% reduction as compared to the 48.4% reduction in the case of IGFBP-1a. This difference in their inhibitory effects is statistically significant (p<0.05). At 20 nM, IGFBP-1b appeared to have a weaker effect compared to IGFBP-1a (IGFBP-1a, 184.1±10.1% vs. IGFBP-1b, 259.6±12.7 of the control group). These data suggest that both IGFBP-1a and -b are functional and both inhibit zebrafish embryo growth and development by binding to IGFs. However, the two proteins are clearly not identical functionally. IGFBP-1a has a stronger activity than IGFBP-1b in inhibiting IGF actions.

## Discussion

In this study, we have identified a pair of co-orthologs of human IGFBP-1 in zebrafish and examined their gene structures, chromosomal locations, spatial and temporal expression patterns, physiological regulatory mechanisms, ligand binding kinetics, and biological activities. The conclusion that zebrafish *igfbp-1b* is a human IGFBP-1 co-ortholog is well supported by phylogenetic and detailed structural data. For instance, zebrafish IGFBP-1b has no cysteine residue in its L-domain, while all known IGFBP-4s have 2 cysteine residues in the L-domain [18]. It is known that mammalian IGFBP-4, but not IGFBP-1, is cleaved by a specific protease, pregnancy associated plasma protease (PAPP)-A, and that the PAPP-A cleavage site is located in the L-domain of all known IGFBP-4s [19], [20], [21], [22]. The new IGFBP lacks the basic residues critical for PAPP-A recognition in the corresponding region. In many mammalian species, IGFBP-1 and -3 genes are located next to each other on the same chromosome in a tail-to-tail orientation. By physical mapping and database searching, we found that zebrafish *igfbp-1a* and *igfbp-3* are neighboring genes located on LG20 in a tail-to-tail orientation, resembling the situation of human *IGFBP-1* and *-3* genes localized on chromosome 7 [16], [17]. If zebrafish *igfbp-1a* and *igfbp-1b* arose by a chromosomal duplication event, one would expect that zebrafish *igfbp-1b* should also be located next to another *igfbp-3* gene. Indeed, analyzing sequences adjacent to *igfbp-1b* on LG2 reveals sequences with significant identity with fish and human IGFBP-3 (data not shown). Whether these sequences indeed encode a functional zebrafish *igfbp-3* awaits further molecular and functional study. In addition to these IGFBP genes, we were able to identify a number of orthologous genes between zebrafish LG2 and human Chr.7. These synteny relationships strongly suggest that zebrafish *igfbp-1a* and *igfbp-1b* are co-orthologs of the human *IGFBP-1* gene and that they originated from a gene duplication event that occurred prior to the teleost radiation.

The finding that two *igfbp-1* genes are present in the zebrafish genome raised the interesting question regarding their functional relationship. After genome duplication, each gene copy can follow a separate evolutionary fate. It has been postulated that non-functionalization is the fate for the majority of the duplicate genes and only 20% or so are retained [23], [24]. Indeed, when the putative zebrafish orthologs of 74 human genes on chromosome 17 were examined, only 15 (20%) were present as duplicate genes [25], [26]. Subfunction partitioning is common among the retained duplicated genes [5]. Despite a large list of examples, in only a few cases has the subfunction partitioning of mammalian gene functions by zebrafish duplicates been explained on a molecular level [5]. For example, differential temporal and/or spatial expression profiles have been documented in duplicated zebrafish *hox1a/b* and *sox9a/b* [27], [28], [29], [30]. In those cases, it has been postulated that the degenerative loss of regulatory modules in each of the two duplicates may have been sufficient to allow the preservation of the two genes, such that the duplicates together retain original functions of the single ancestor gene [5], [31]. The duplicated zebrafish *igfbp-1* genes are clear examples of temporal subfunctioning partitioning. In humans and mouse, IGFBP-1 is expressed only in the liver [11]. The zebrafish paralogs are also expressed exclusively in the liver in adult fish. During embryogenesis, the two genes are expressed in overlapping spatial domains. The two genes, however, have distinct temporal patterns. While zebrafish IGFBP-1a mRNA was easily detected throughout embryogenesis, IGFBP-1b mRNA was not detectable or was present at very low levels in early stages. It is only highly expressed in advanced embryonic stages.

In humans and mice, IGFBP-1 is highly induced by a variety of catabolic conditions, including fasting, malnutrition, and endoplasmic reticulum stress [11]. Another major physiological cue that regulates IGFBP-1 expression is hypoxia. Hypoxia triggers the induction of the human IGFBP-1 gene via the hypoxia-inducible factor-1 (HIF-1) complex [32]. The results of this study indicate that fasting and hypoxia induce the hepatic expression of the two zebrafish IGFBP-1 co-orthologs. One of the interesting findings in the present study is while both *igfbp-1a* and *igfbp-1b* are hypoxia-inducible, their timing and responsiveness to hypoxia are clearly different. In early embryonic stages, hypoxia strongly induces the *igfbp-1a* expression in early embryogenesis, whereas it represses *igfbp-1b* expression. In advanced embryonic stages, it induced a marked increase in *igfbp-1b* expression, while the same treatment had no effect on the levels of *igfbp-1a* expression. In adult fish, hypoxia has a stronger effect on *igfbp-1a* than on *igfbp-1b* expression, suggesting that the duplicated genes underwent subfunction partitioning. We term this type of subfunction partitioning as inducible or regulation subfunctioning partitioning as it is distinct from the simply spatial, temporal, qualitative, and quantitative partitioning previously reported [5].

The differential responses of *igfbp-1a* and *igfbp-1b* to hypoxia are likely due to changes/mutations in their *cis*-regulatory elements after the duplication. The HIF-1 complex activates target gene expression by direct binding to the hypoxia-response element (HRE; A/G CGTG) [33]. Tazuke et al. (1998) have identified a HRE located in intron 2 of the human IGFBP-1 gene responsible for its hypoxia response [32]. We have recently shown that the induction of zebrafish *igfbp-1a* expression by hypoxia is mediated through HIF-1 both *in vitro* and *in vivo* [34]. The zebrafish *igfbp-1a* promoter contains 13 potential HREs within a 2.1 kb region upstream of the translational start site and the HRE positioned at −1090/−1086 is primarily responsible for the hypoxia and HIF-1-induced IGFBP-1 expression. An additional *cis* element, HIF-1 ancillary sequence (HAS), is required for the responsiveness to hypoxia and to HIF-1 [34]. In this study, we show that there are 5 putative HREs and 3 possible HASs in the 2.3 kb region upstream of the translational start site of zebrafish *igfbp-1b*, indicating possible re-arrangement of these cis elements in the duplicated gene. This subfunction partitioning of IGFBP-1 genes provides an excellent opportunity to identify critical regulatory elements. Further functional studies will undoubtedly reveal the factors responsible for the unique expression patterns of these two genes.

Subfunction partitioning can involve protein structural changes and has important functional and evolutionary consequences, but few such examples have been established in zebrafish. In this study, we provide biochemical and biological evidence indicating subfunction partitioning involved protein structural and functional changes in the duplicated *igfbp-1* genes. We show that purified zebrafish IGFBP-1a and -1b proteins have different apparent molecular mass on SDS-PAGE. A single *N*-glycosylation motif, that could account for the larger molecular mass, is found in IGFBP-1a but not -1b. While both zebrafish IGFBP-1a and -1b are capable of IGF binding, binding kinetics analysis revealed that the two proteins have very different IGF binding properties. Zebrafish IGFBP-1a has very high-affinity for IGF-I (*K_D_* = 0.28 nM) and IGF-II (*K_D_* = 0.34 nM) and the IGFBP-1a/IGF complexes are highly stable. In comparison, the affinities of IGFBP-1b to IGF-I and IGF-II are 43 and 27 fold lower. Interestingly, IGFBP-1b retains a remarkably high association rate with IGF-I and IGF-II but the IGFBP-1b/IGF complexes are much less stable, due to the high dissociation rates. In mammalian IGFBPs, amino acid residues responsible for IGF-binding have been elucidated [35]. Further mutational analysis of zebrafish IGFBP-1a and -1b will undoubtedly provide structural insights underlying the different ligand binding properties of the two zebrafish IGFBP-1 proteins.

Studies in a variety of species have shown that the IGF signaling pathway plays a fundamental role in regulating wide range of physiological events from embryonic development and growth to life span regulation [12], [13]. The multiple forms of IGFBPs bind the IGF ligands and regulate the availability of the IGFs to the IGF-1 receptors, therefore providing additional layers of regulation of the IGF signaling. IGFBP-1 is highly inducible under a variety of catabolic conditions such as food deprivation, malnutrition, stress, injury, endoplasmic reticulum stress, and hypoxia [11]. Recent *in vivo* studies have indicated that the induced IGFBP-1 serves as a molecular switch by restricting IGF signaling and diverts the limited energy resources away from growth and development toward those metabolic processes essential for survival [8]. A number of *in vitro* studies using transgenic mice have shown that IGFBP-1 has an inhibitory effect on IGF activity [36], [37], [38], [39], [40], [41]. The inhibitory effects seem to be mainly caused by competition for IGF binding with the IGF-1R [11]. In good agreement with these findings, injection of IGFBP-1a and -1b mRNA into zebrafish embryos caused significant decreases in body size and somite number. These overexpression experiments suggest that IGFBP-1a and -1b are inhibitory proteins and that they probably act by binding to and inhibiting IGF actions in vivo. However, the two proteins are clearly not identical in terms of biological potency. When IGFBP-1a or -1b was added to cultured zebrafish embryonic cells in the presence of absence of IGF-I, IGFBP-1b has a significantly lower activity in inhibiting IGF actions compared to that of IGFBP-1a. This is consistent with the fact that zebrafish IGFBP-1b has a much lower affinity for IGF-1 and IGF-II.

**Figure 7:**
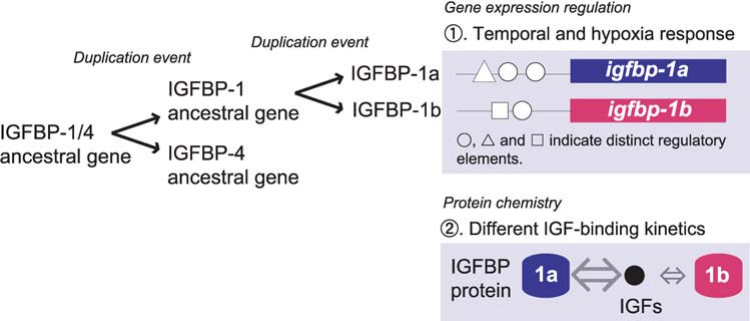
A proposed model for the evolution of the paralogous IGFBP-1 genes in zebrafish. The duplicated IGFBP-1 genes have distinct functions through evolving: 1) differential regulatory mechanisms of gene expression during development, 2) different protein structure and IGF binding kinetics, and 3) different biological activities. These features of the duplicated IGFBP-1 genes would allow greater flexibility and fine-tuning of IGF actions under various physiological conditions during development.

In conclusion, we have identified and characterized a novel zebrafish IGFBP gene, *igfbp-1b*, which is a co-orthologous gene of human IGFBP-1. The duplicated IGFBP-1 genes have evolved overlapping yet distinct biological properties through differentially changing: 1) temporal expression pattern and hypoxia responsiveness and timing, 2) protein structure modules that alter their IGF binding kinetics, and 3) different biological activities (Fig. 7). These distinct features of the duplicated IGFBP-1 genes could allow greater flexibility and fine-tuning of IGF actions under various physiological conditions during development.

## Materials and Methods

### Chemicals

All chemicals and reagents were purchased from Fisher Scientific (Pittsburgh, PA) unless noted otherwise. RNase-free DNase and restriction enzymes were purchased from Promega (Madison, WI). Taq DNA polymerase and Vent DNA polymerase were purchased from New England Biolabs (Beverly, MA). *PfuTurbo* DNA polymerase were purchased from Stratagene (La Jolla, CA). Superscript II reverse transcriptase (RT) and oligonucleotide primers were purchased from Invitrogen Life Technologies, Inc. (Carlsbad, CA). Oligonucleotide primers for PCR were purchased from Invitrogen. iQ SYBR Green Supermix was from Bio-Rad (Hercules, CA).

### Animals and experimental procedures

Wild-type zebrafish (*Danio rerio*) were maintained on a 14 h light −10 h dark cycle at 28 C and fed twice daily. Embryos were obtained by natural crossing. Fertilized eggs were raised in embryo medium at 28.5 C and staged according to the standard method [42]. To prevent pigmentation, embryos were kept in embryo medium supplemented with 0.003% (w/v) 2-phenylthiourea beginning at 10 h post fertilization (hpf). In the fasting experiments, adult zebrafish were fasted for 1, 2, or 3 week after 1 week of acclimation. After 2 week of fasting, the remaining fish were divided into two groups. The first group continued food deprivation, while the other group was re-fed for 1 week. In the hypoxia experiments, oxygen was depleted by bubbling water with nitrogen gas. Oxygen concentrations were measured using a dissolved oxygen meter (YSI Model 58, Fisher Scientific, Pittsburgh, PA). The dissolved oxygen content in the hypoxia group was set as 0.67±0.06 mg/l for embryos and 1.85±0.16 mg/l for adult fish, whereas the normal ambient oxygen levels were 6.6±0.45 mg/l. Fish were exposed to hypoxia for 24 h (embryo) or 6 h (adult fish) at 28.5 C. All experiments were conducted in accordance with the guidelines established by the University Committee on the Use and Care of Animals at the University of Michigan.

### Molecular cloning and physical mapping

Using the human IGFBP-4 amino acid sequence as a query, we searched the zebrafish genome database (Ensembl Multi Blast view: http://www.ensembl.org/Multi/blastviewspeciesDanio_rerio), and found a putative IGFBP gene (ENSDARG00000038666). The full-length sequence of this putative IGFBP was determined by 5′- and 3′- rapid amplification of cDNA ends (RACE) utilizing the SMART RACE kit (Clontech, Mountain View, CA). Phylogenetic analysis was done using full-length amino acid sequences by Maximum Likelihood method. In the PlotTest program, the best model (JTT+G+I) was selected and the tree was made based on PHYML program. Amino acid sequences used for this study are summarized in supplemental table (Table S1) with their accession numbers or reference numbers. Synteny analysis was carried out using NCBI Map Viewer based on *Danio rerio* Zv6 and *Homo sapiens* Build 36.3 (http://www.ncbi.nlm.nih.gov/mapview/) and data from zebrafish and human synteny map [43]. Genomic structure was determined by searching the zebrafish genome (http://http://www.ensembl.org/Multi/blastview?species=Danio_rerio) followed by PCR analysis. The LN54 radiation hybrid panel was used for physical mapping [44].

### Reverse transcription (RT)-PCR and quantitative real-time RT-PCR (qRT-PCR)

Total RNA was isolated from adult zebrafish and embryos. After DNase treatment, total RNA (500 ng) was reverse-transcribed to single strand cDNA using SuperScript II reverse transcriptase according to the manufactures instructions. RT-PCR was performed with a set of primers (5′-CTGAGCTTCTCGCGTCTCTGGACGTCATCACAGAG-3′ and 5′-TCAGTGTGTGAGCTCCTGTGGGCA-3′) using Taq-DNA polymerase. The signal intensity of RT-PCR products was measured densitometrically using Image J software (NIH, Bethesda, MD), and normalized by β-actin levels.

qRT-PCR was carried out on iCycler iQ Multicolor real-time PCR detection system (Bio-Rad) according to the manufacturer's instructions. Primer sequences for real-time qPCR were β-actin: 5′-AGGTCATCACCATTGGCAAT-3′ and 5′-GATGTCGACGTCACACTTCAT-3′; IGFBP-1a: 5′-CCACCAGTTTCTTGCGTATC-3′ and 5′-AGCCTAACCACAGCCAAAGCGAG-3′; and IGFBP-1b: 5′-ACCACCCTACTGAAGAGGACACAGA-3′ and 5′-GCGTTGAGTTGTGACTTGATGCTC-3′. qPCR was performed using iQ SYBR Green Supermix (Bio-Rad). One µl of cDNA product was used as PCR template. Plasmid cDNAs for IGFBP-1a, 1b, and β-actin, a housekeeping gene, were used as controls. Each assay for unknown samples was performed simultaneously with standard samples and negative control samples all in triplicate wells in the same plate. Standard curves constructed from serial dilution of standard plasmids were analyzed. The number of molecules of a particular gene transcript was calculated based on the standard curve and normalized to the β-actin mRNA level. The specificity of the PCR was verified by denaturing curve analysis and direct sequencing of the products.

### Whole mount in situ hybridization

The plasmid containing IGFBP-1b cDNA (746 bps) was linearized by restriction enzyme digestion, followed by *in vitro* transcription reactions with either T7 or T3 RNA polymerase, to generate digoxigenin (DIG)-labeled RNA probes using an mMessageMachine kit (Ambion, TX). The specificity of the riboprobe was verified by dot-blot analysis and it did not cross-react with full-length IGFBP-1a cDNA. Hybridization was carried out as described previously [7]. Images were captured with a Nikon DC50NN camera mounted to a Nikon Eclipse E600 microscope (Melville, NY).

### Expression and purification of recombinant IGFBP-1 proteins

Recombinant *Myc*-tagged and 6×Histidine-tagged IGFBP-1a, IGFBP-1b, and human IGFBP-1 were produced in human embryonic kidney (HEK) 293t cells. Briefly, cells were cultured in high glucose Dulbecco's modified Eagle's medium supplemented with 10% fetal bovine serum (FBS), 2 mM glutamine, and non-essential amino acids (Life Technologies). Cells were transfected with various expression plasmids. The transfected cells were grown in selection medium containing 1.75 mg/ml Geneticin (G418). After selection, cloned cells were pooled and maintained in complete medium containing 0.5 mg/ml G418. Confluent cells were washed and conditioned in serum-free medium (SFM) for 48 h. The expression of target protein was confirmed by Western blotting using a monoclonal anti *c*-*myc* antibody (Santa Cruz, CA). Nickel-chelate affinity resin (Ni-NTA agarose, Invitrogen) was used for affinity purification following manufacturer's instruction. Bound protein was eluted with 250 mM imidazol in PBS containing 2.5 M NaCl and 250 mM NaH_2_PO_4_, pH 8.0. To ensure the absence of IGF-I or -II in the preparation of IGFBP-1s, affinity-purified material (100 µg) was further loaded onto a Superdex G-75 column (Amersham), equilibrated, and eluted with 50% formic acid at 0.5 ml/min. At the acidity of the solvent (pH<1), bound IGF should dissociate from IGFBP and elute separately [45]. The purified protein was ultrafiltered, dissolved in water, and lyophilized. After the purity was determined by silver staining, the purified protein was quantified using a BCA protein assay kit (Pierce Biotechnology, Rockford, IL). Also, IGF binding ability was confirmed by western-ligand blot analysis by utilizing DIG-labeled IGF-1 according to previous study [46]. The recombinant human IGFBP-1 was purified by affinity chromatography using a metal chelate column (NTA-Sepharose, GE Healthcare) followed by reversed-phase High Pressure Liquid Chromatography (RP-HPLC) on a column packed with Nucleosil C4 500-7 (Macherey-Nagel). The purified protein was lyophilized and dissolved in 10 mM HEPES, 150 mM NaCl, 3 µM EDTA, 0.05% TWEEN-20, pH 7.4 (HBS).

### Surface plasmon resonance analysis

Surface plasmon resonance experiments were carried out on a BIAcore T100 instrument (BIAcore AB, Uppsala, Sweden) using series S CM5 sensor chips and coupling reagents supplied by the manufacturer. Immobilization of human IGF-I and -II (Diagnostic Systems Laboratories) was performed by first activating the surface of the sensor chip with a mixture of EDC/NHS followed by injection of IGF-I or -II (10 µg/ml in 10 mM sodiumacetate, pH 4.75). Remaining activated groups were blocked by injection of 1.0 M ethanolamine. The levels of immobilization were 120 RU and 138 RU for IGF-I and -II, respectively. All binding experiments were carried out at 25 C in HBS with a constant flow rate of 30 µL/min. Different concentrations of IGFBP-1s were injected over the surface for 60 sec followed by a five minute dissociation period. The sensor surface was regenerated by two 30 sec injections of HCl (100 mM). Sensorgram data were analyzed using the BIAcore T100 evaluation Software version 1.1. A 1∶1 model was globally fitted to the sensorgram data to obtain *k_a_* and *k_d_* values for the interaction. The equilibrium dissociation constant K_D_ was subsequently calculated as the ratio *k_d_/k_a_*. The reliability of kinetic analysis was shown by lower χ-square value (χ^2^<5.0).

### Microinjection experiment

Capped mRNAs of EGFP, zebrafish IGFBP-1a, and -1b were synthesized by *in vitro* using the mMessage mMachine kit (Ambion, TX). Microinjection volume was estimated at 1 nl/embryo, based on calibrations using known quantities of solution. EGFP mRNA (600 pg/embryo) or a mixture of IGFBP mRNA (400 pg/embryo) and EGFP mRNA (200 pg/embryo) was injected to embryos at 1–2 cell stage. Body length and somite number were measured at 24 hpf as described previously [8].

### Cell culture and BrdU labeling assay

Zebrafish embryonic cells (ZF-4) were plated onto coverslips placed in 6-well plates and grown to 80–100% confluence. After 20 h serum starvation, cells were exposed to 20 µM 5-bromo-2′-deoxyuridine (BrdU) with or without 20 nM of IGF-I. Various concentrations of IGFBP-1s (2, 10, 20 or 50 nM) were added together with IGF-I to examine the effects of IGFBP-1s on IGF activities. To evaluate possible IGF-independent effect, cells were treated with 50 nM of IGFBP-1s alone. The percentage of BrdU-positive cells in each group was determined as reported previously [47].

### Statistics

Statistical significance among experimental groups was determined by one-way analysis of variance (ANOVA), followed by the Newman-Keuls multiple comparison test. All values are represented as means±standard error (S.E.). Calculation was performed using GraphPad Prism 3.0 software (GraphPad Software Inc., San Diego, CA), and significance was accepted at p<0.05 or better.

## Supporting Information

Table S1 (0.07 MB DOC)
