# Supplementary material for: Duplication and Diversification of the Hypoxia-Inducible IGFBP-1 Gene in Zebrafish
Source: PLoS One. 2008 Aug 28;3(8):e3091. doi: 10.1371/journal.pone.0003091 (PMC2518108; doi:10.1371/journal.pone.0003091)
Supplement: Table S1 — (0.07 MB DOC) [file pone.0003091.s001.doc]

Table S1. Accession numbers of IGFBP sequences used for sequence analyses in this study

| †Species / Protein | IGFBP-1 | IGFBP-2 | IGFBP-3 | IGFBP-4 | IGFBP-5 | IGFBP-6 |
| --- | --- | --- | --- | --- | --- | --- |
| human | NP_000587 | AAA36048 | P17936 | NP_001543 | AAA53505 | NP_002169 |
| mouse | P47876 | P47877 | NP_032369 | NP_034647 | NP_034648 | NP_032370 |
| rat | P21743 | NP_037254 | NP_036720 | NP_001004274 | NP_036949 | NP_037236 |
| pig | Q75ZP3 | NP_999168 | P16611 | - | NP_999264 | - |
| bovine | P24591 | NP_776980 | NP_776981 | AAB24873 | NP_001098797 | NP_001035585 |
| chicken | NP_001001294 | NP_990690 | - | NP_989684 | - | - |
| frog | NP_001082206 | - | - | ENSXETG00000021361 | NP_001083938 | - |
| salmon | AAV83995 | - | - | - | - | - |
| trout | ABA55020 | ABA33956 | NP_001118029 | ABA33955 | NP_001118124 | ABA55019 |
| fugu | NEWSINFRUG00000160007 NEWSINFRUG00000121680 NEWSINFRUG00000157334 | NEWSINFRUG00000164876 NEWSINFRUG00000122413 | NEWSINFRUG00000134657 | NEWSINFRUG00000145668 | NEWSINFRUG00000142978 | - |
| tetraodon | GSTENG00026400001 | GSTENG00016851001 GSTENG00015886001 | GSTENG00004782001 GSTENG00012277001 | - | GSTENG00016850001 | GSTENG00004132001 |
| zebrafish | NP_775390 | NP_571533 | NP_991314 | - | NP_001119935 | - |

†: human (*Homo sapiens*), mouse (*Mus musculus*), rat (*Rattus norvegicus*), pig (*Sus scrofa*), bovine (*Bos taurus*), chicken (*Gallus gallus*), frog (*Xenopus laevis*), salmon (*Oncorhynchus tshawytscha*), trout (*Oncorhynchus mykiss*), fugu (*Takifugu rubripes*), tetraodon (*Tetraodon nigroviridis*), zebrafish (*Danio rerio*)
